# Enriched Environment Facilitates Anxiolytic Efficacy Driven by Deep-Brain Stimulation of Medial Prefrontal Cortex

**DOI:** 10.3389/fnbeh.2018.00204

**Published:** 2018-10-09

**Authors:** Yamini Bhaskar, Lee Wei Lim, Rupshi Mitra

**Affiliations:** School of Biological Sciences, Nanyang Technological University, Singapore, Singapore

**Keywords:** anxiety, complex housing, plasticity, ventromedial prefrontal cortex, morphology, neurons

## Abstract

Deep brain stimulation (DBS) is a widely used treatment for neurodegenerative disorders like Parkinson’s disease. Recently, several studies have used preclinical animal models to suggest that DBS has a potential to improve emotional symptoms in mental disorders such as treatment-resistant depression and post-traumatic stress disorder. An important difference between neurodegenerative and emotional disorders is the crucial role of environment in the ontogeny of the latter. Thus, it is important to understand the effects of DBS in the context of environmental variation. In this study, we show that DBS of ventromedial prefrontal cortex reduces anxiety in rats when it is coupled with simultaneous exposure to an enriched environment (EE). In contrast, effects of DBS on anxiety-like behaviors remained equivocal when animals were housed in standard laboratory conditions. These results suggest that the ability of DBS to treat anxiety and related phenotypes can be significantly enhanced by EE opportunities.

## Introduction

Deep brain stimulation (DBS) is a surgical therapeutic approach for treating disorders of the central nervous system. It uses electrodes implanted into anatomically defined brain targets to deliver current stimulation in a chronic manner (Kühn and Volkmann, 2017). It has been previously used as an effective treatment in movement disorders like Parkinson’s disease (Hickey and Stacy, 2016) and several psychiatric disorders like Tourette syndrome and obsessive-compulsive disorder (Alonso et al., 2015; Fraint and Pal, 2015). This success has led to suggestions that this technique can also be used in treatment-recalcitrant cases of other brain-centric pathologies like post-traumatic stress disorder (Reznikov et al., 2016) or major clinical depression (Torres-Sanchez et al., 2017).

The DBS of the ventromedial prefrontal cortex in rats reduces learned helplessness in a forced swim task (Hamani et al., 2010). Stimulation of this brain region also rescues anhedonia caused by chronic unpredictable stress, as evidenced by the preference of sucrose over water (Hamani et al., 2012). Similarly, ventromedial prefrontal cortex stimulation rescues endophenotypes related to anhedonia and learned helplessness in a mouse strain selectively bred to show greater depression-like behaviors (Schmuckermair et al., 2013). Stimulation of the same brain region also rescues depression-like behaviors resulting from olfactory bulbectomy in rats (Jiménez-Sánchez et al., 2016). Human subcallosal cingulate gyrus is thought to be an analogous structure to rodent ventromedial prefrontal cortex. Rodent preclinical studies are, in fact, subsequent to several clinical case studies and open-label studies suggesting improvement of depressive symptoms after DBS in subcallosal cingulate gyrus (reviewed in Dandekar et al., 2018). While some of the follow-up randomized controlled design studies confirm therapeutic benefits of DBS (Eitan et al., 2018), other studies do not show significant improvement (Holtzheimer et al., 2017; Merkl et al., 2018).

Other brain structures have also been examined as a target for DBS in treatment-refractory depression (Dandekar et al., 2018). Similarly, several brain regions have been examined as targets for lowering anxiety and depression-like behaviors. This includes the nucleus accumbens, ventral tegmental area, lateral habenula, subthalamic nucleus and medial forebrain bundle (Dandekar et al., 2018). Stimulation of ventromedial prefrontal cortex among these varied structures shows the most robust reversal of anxiety in stress-naïve animals; and reduction of stress-induced depressive and anxiety-like behaviors (Lim et al., 2015). Various brain structures have also been used as sites for DBS in animal models of fear and anxiety (Reznikov et al., 2016). This includes basolateral amygdala, prefrontal cortex, striatum and hippocampus. The choice of these brain structures in preclinical models arises from their role in forming brain circuits for fear learning and extinction. Among these structures, we have used DBS in ventromedial prefrontal cortex, showing that stimulation of this brain region also improves cognition in middle-aged rats (Liu et al., 2015). Thus, stimulation at the ventromedial cortex provides continuity with the previous work, while showing promise in preliminary clinical work. In this backdrop, we chose the ventromedial prefrontal cortex as the stimulation site in the current study.

In short, preclinical work shows promising results suggesting that DBS of the ventromedial prefrontal cortex can reduce anxiety and depression. Yet, the ontogeny of emotional disorders, for example, depression or consequences of prior trauma, is intricately linked to environmental factors. This contrasts with neurodegenerative disorders more commonly targeted by DBS. For example, a genetic predisposition to depression in both cohorts only manifests itself after exposure to childhood adversity (Caspi et al., 2003). Animal work congruently demonstrates the crucial role of environment in emotional behaviors. Stimuli that show robust anxiogenesis in a sparse housing environment fail to generate anxiety in complex housing regimes (Ashokan et al., 2016, 2018a; Koe et al., 2016) or when the ambient quality of the environment is changed (Abdulai-Saiku et al., 2017). Sensory enrichment of the housing environment induces robust structural changes in brain regions important for emotional behaviors including the ventromedial prefrontal cortex (Ashokan et al., 2018b), hippocampus (Darmopil et al., 2009), and basolateral amygdala (Ashokan et al., 2016; Koe et al., 2016). These studies collectively show that symptoms of emotional disorders are contingent on the environment of the individuals. Hence, the preclinical work for brain stimulation, when in the context of emotional plasticity, must also encompass interactions of the stimulation with the ambient environment of the animals. Housing conditions are known to influence anxiety and underlying plastic changes in the neuronal architecture. Thus, changes in the housing environment are a promising avenue to increase the efficacy of therapeutic changes in behavior brought about by DBS. This possibility has not yet been tested. In the present study, we attempt to bridge this gap by delineating if the efficacy of ventromedial prefrontal cortex DBS on anxiety-like behaviors depends on the housing environment of the rats. Specifically, we ask if ameliorating effects of DBS on anxiety can be enhanced by housing in an enriched environment (EE).

## Materials and Methods

### Animals and Experimental Design

Adult Wistar male rats (average age: 8 weeks, average weight: 250 g) were used for this experiment. Animals were housed in reversed day–night cycle (lights on at 19:00 h) and *ad libitum* access to food and water. Animals were handled everyday to get them used to human handling to prevent stress during the behavior trials. All experimental procedures were reviewed and approved by the Institutional Animal Care and Use Committee (IACUC) of NTU. All experiments were performed in accordance with IACUC guidelines and regulations.

Animals were randomly divided to receive either sham treatment or DBS to ventromedial prefrontal cortex. Both sham and stimulated animals were further subdivided and housed in either standard laboratory housing or enriched housing. Before assignment to experimental groups, all animals stayed in standard housing conditions postweaning (2/cage).

### Housing Conditions

Standard laboratory housing consisted of two animals living in an animal facility cage (37 × 22 × 18 cm). Animals in the standard group were singly housed after surgery for electrode implantation. This was done to prevent damage to the electrode site due to physical interaction with cage mates. EE consisted of larger cages (72 × 51 × 110 cm), more animals per cage (four animals per cage), and presence of novel objects. Larger spaces within enriched housing prevented electrode damage during group housing. The novel objects included climbing walls made of wire-net, plastic tunnels, plastic and wooden objects of varied colors and textures, ample nesting material and gustatory variety in the form of fruit loops and sunflower seeds and layered tiers within the cage. Running wheel was not provided in the EE to minimize effects of exercise after recent surgery. The arrangement of the objects was changed every fourth day. Animals were placed in enriched housing from day 1 (surgery) to day 19 (sacrifice). Animals assigned to enriched housing were housed 2/cage in standard housing conditions before the surgery.

### DBS

All animals were implanted with an electrode directed at the ventromedial prefrontal cortex under general anesthesia achieved by a cocktail of ketamine and xylazine. The plane of anesthesia was maintained using gaseous isoflurane during stereotaxic surgery (2.5% v/v). The rat was positioned and fixed in a standard stereotactic apparatus. A midline incision was made from the orbital level to the occipital lobe, which allowed adequate exposure to the skull. A burr hole was made above the anatomical target followed by duratomy. Miniature screws (0.8 mm; 2 per hemisphere) were placed into the skull anteriorly and posteriorly to the burr holes to serve as anchors for cement. Electrodes were implanted into the ventromedial prefrontal cortex and fixed with dental cement that adhered to the electrode construction and miniature screws (AP: +2.70 mm; L: ±0.60 mm; V: 4.60 mm). Bipolar stimulating electrodes (Synergy, Singapore) with an inner platinum–iridium core wire with a gold-plated cannula were used (Technomed, Beek, Netherlands). Finally, the skin was carefully repositioned and stitched up. Experiments started after 10 days from surgery.

A digital stimulator DS8000 and stimulus isolators DLS100 (World Precision Instruments, Sarasota, FL, USA) were used to deliver the electrical stimuli in animals assigned to the DBS group. Pulse width of the stimulation was set at 100 μs and amplitude at 200 μA. Animals received stimulation for 1 h daily from day 11 to day 19 (100 Hz, surgery being day 1). Sham animals were brought to the stimulation room for the duration of stimulation protocol and connected to the stimulator, but no stimulation followed.

### Behavioral Testing

All behavioral tests were conducted between 08:00 h and 12:00 h under dim red lights. Animals were allowed to habituate to the testing room for >30 min before the test. One day passed between successive behavioral tests in the sequence of home-cage emergence assay (day 11), object recognition task (days 13 through 15), and elevated plus-maze (day 16). Behavioral arenas were cleaned with 70% ethanol in-between trials.

### Home Cage Emergence

A rat in its home cage was moved from the holding room to the test room. After habituation, the home-cage was left open, and the rat was offered the possibility of emerging via a grid. This was observed for 5 min. The latency to emerge from the home cage (i.e., the time until the rat was on the grid outside its home cage with all four legs) was scored. Trial duration was 300 s. A score of 300 was arbitrarily assigned to any animal which did not emerge for the duration of the trial.

### Elevated Plus-Maze

Anxiety-like behavior was measured using an elevated plus-maze that consisted of a plus-shaped arena with two open (75 × 11 cm, 1 cm wall, 3–4 lux illumination) and two enclosed arms (75 × 11 cm, 26 cm wall, 0 lux illumination). The arena was elevated to 60 cm above the ground. The animal was placed at the center at the start of the trial (trial duration = 300 s). Exploration in open and enclosed arms was quantified. Open arm exploration (entries and occupancy time) relative to the sum of open and enclosed arm explorations was used as an index for anxiety. Mean of percentage open arms entries and percentage open arms time was subtracted from 100 to derive an index for anxiety. Entry in an arm was defined as the presence of the whole body including head, four paws, and at least the base of tail inside the open arm. Also, the number of head dips was quantified as a measure of risk assessment in the maze. Head dip was defined as downward movement of the head toward the floor, extending completely out of the open arm.

### Object Recognition Task

The rats were introduced to a square arena (1 m × 1 m) with opaque walls. Two similar objects (1-liter laboratory glass bottles) were diagonally placed in the arena. Animals were allowed to explore the arena for 180 s. Short-term object recognition memory was tested 90 min afterward. One of the previously presented objects was replaced with a rectangular box during this phase. Exploration of novel and familiar objects was quantified over a period of 180 s. Object recognition was quantified as exploration of novel objects relative to the sum of exploration for novel and familiar objects.

### Statistical Analysis

GraphPad Prism version 7 was used for statistical analysis. Figures represent mean and SEM of rank-transformed data, along with individual values for each animal. Numbers of animals used for analysis are depicted in each figure.

Normality for behavioral endpoints was tested using the Shapiro−Wilk test. Several endpoints exhibited significant departure from normality. Consequently, nonparametric statistics was used for intergroup comparisons (Kruskal–Wallis test). Data for each endpoint were further rank-transformed across four experimental groups.

This study was built to test an *a priori* premise that DBS shows greater clarity and more robust effects when applied in enriched housing rather than standard housing. Congruent with *a priori* assumptions, two planned comparisons were set before data collection started: sham and stimulated animals in standard housing and sham and stimulated animals in enriched housing (Ruxton and Beauchamp, 2008). Orthogonal comparisons were used, such that no experimental group was used in more than one comparison. For example, we did not test statistical significance for effects of enriched housing itself vis-à-vis standard housing in absence of DBS. Independent sample Student’s *t*-test was used for parametric planned comparisons of rank-transformed data. Interpretation of these planned comparisons was buttressed by the calculation of effect size for rank-transformed data using Cohen’s *d* (Lakens, 2013).

## Results

Animals housed in the standard laboratory housing or EE were subjected to either DBS directed at the ventromedial prefrontal cortex or sham stimulation, yielding four experimental groups.

### The EE and DBS Decreased Latency to Emerge From the Home-Cage

**Figure 1:**
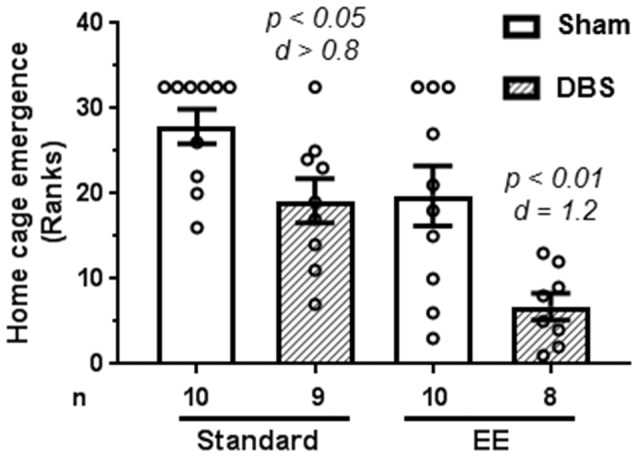
Effects of deep brain stimulation (DBS) on latency to emerge from home-cage in presence or absence of enriched environment (EE). Emergence latency (in seconds) was rank-transformed for the illustration. Mean and SEM, along with individual rank of animals in each group, are depicted. Number of animals (*n*) is also presented. Both *p* and Cohen’s *d* values are shown for statistically significant comparisons between DBS and corresponding sham groups.

A Kruskal–Wallis test revealed the presence of statically significant intergroup differences (H_4_ = 17.4, *p* = 0.0006) in latency to emerge from home-cage. The data were rank-transformed and analyzed using planned comparisons. The DBS reduced emergence latency in animals housed in both standard housing (Figure 1; *t*_(17)_ = 2.66, *p* = 0.016) and EE (Figure 1; *t*_(16)_ = 3.07, *p* = 0.007). However, effects of DBS were more pronounced in presence of EE (Cohen’s *d* = 1.19; rank difference = 13.0 ± 3.8) compared with absence of EE (Cohen’s *d* = 0.82; rank difference = 8.7 ± 3.7).

### DBS Reduced Anxiety-Like Behavior in Elevated Plus-Maze When Animals Were Placed in Enriched Housing

Anxiety-like behavior was quantified using reduction in anxiogenic open arms as a proxy. Open arm exploration was defined as the mean of the percentage of open arm entries and percentage of time spent in the open arm. Anxiety was defined as hundred minus percentage of open arm exploration.

**Figure 2:**
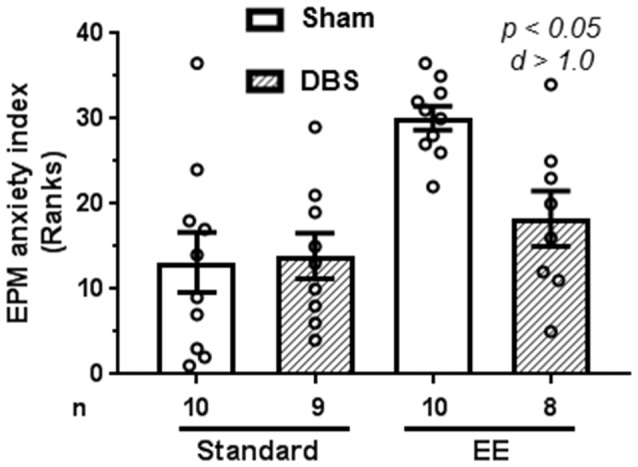
Effects of DBS on anxiety in an elevated plus-maze in presence or absence of EE. Anxiety index was expressed as subtraction of open-arm exploration from 100. This endpoint was rank-transformed for the illustration. Mean and SEM, along with individual rank of animals in each group, are depicted. Number of animals (*n*) is also presented. Both *p* and Cohen’s *d* values are shown for statistically significant comparisons between DBS and corresponding sham groups.

A Kruskal–Wallis test revealed presence of statically significant intergroup differences (H_4_ = 15.4, *p* = 0.0015) in anxiety-like behavior. Analysis of rank-transformed data revealed that DBS reduced anxiety-like behavior in animals housed in EE (Figure 2; *t*_(16)_ = 3.60, *p* = 0.002). In contrast, DBS did not cause significant effects on anxiety-like behavior in animals housed in standard laboratory cages (Figure 2; *t*_(17)_ = 0.16, *p* = 0.872). Effects of DBS on anxiety were more pronounced in presence of EE (Cohen’s *d* = 1.01; rank difference = 11.8 ± 4.1) compared with absence of EE (Cohen’s *d* = 0.06; rank difference = −0.7 ± 3.9).

### EE and DBS Increased Risk Assessment in Elevated Plus-Maze

Risk assessment in elevated plus-maze was quantified as number of head dips made during the trial.

**Figure 3:**
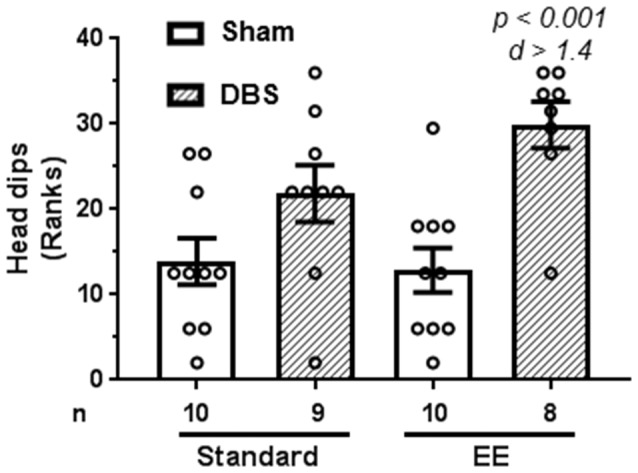
Effects of DBS on risk assessment in an elevated plus-maze in presence or absence of EE. Risk assessment was measured as number of head dips made during the trial. This endpoint was rank-transformed for the illustration. Mean and SEM, along with individual rank of animals in each group, are depicted. Number of animals (*n*) is also presented. Both *p* and Cohen’s *d* values are shown for statistically significant comparisons between DBS and corresponding sham groups.

A Kruskal–Wallis test revealed presence of statically significant intergroup differences (H_4_ = 14.4, *p* = 0.0024) in risk assessment behavior. Planned comparisons for rank-transformed data showed that DBS reduced risk assessment behavior in animals housed in EE (Figure 3; *t*_(16)_ = 4.47, *p* < 0.001). In contrast, DBS did not cause significant effects on risk assessment in animals housed in standard laboratory cages (Figure 3; *t*_(17)_ = 1.87, *p* = 0.079). Effects of DBS on anxiety were more pronounced in the presence of EE (Cohen’s *d* = 1.44; rank difference = −17.3 ± 4.1) compared with absence of EE (Cohen’s *d* = 0.69; rank difference = −7.9 ± 4.0).

### EE and DBS Did Not Affect Memory Performance in Object Recognition Task

Animals were presented with two identical objects at time zero. Short-term object recognition memory was tested after 90 min by presenting animals a choice between a previously presented familiar object and a novel object. Memory was quantified as percentage time spent exploring novel object relative to sum of time spent exploring novel and familiar objects.

**Figure 4:**
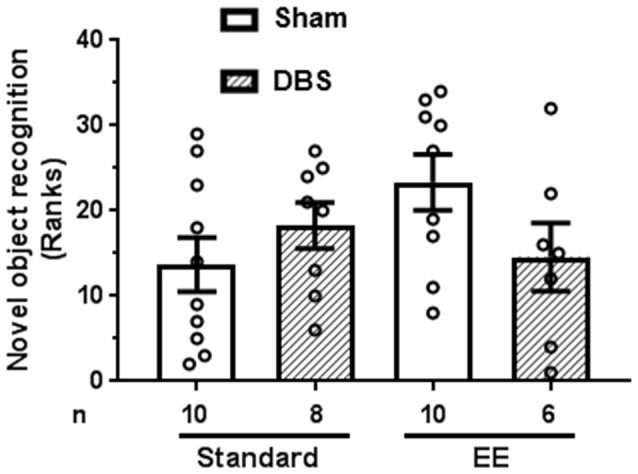
Effects of DBS on short-term memory (90 min) in presence or absence of EE. Object recognition was measured as percentage time spent near novel object relative to total time spent with novel and familiar object (chance = 50%). This endpoint was rank-transformed for the illustration. Mean and SEM, along with individual rank of animals in each group, are depicted. Number of animals (*n*) is also presented.

A Kruskal–Wallis test revealed absence of statistically significant intergroup differences in short-term memory (H_4_ = 5.2, *p* = 0.158). The data were rank-transformed and analyzed using planned comparisons. Sidak’s multiple comparisons test did not reveal statistically significant effects of DBS in presence (Figure 4; *t*_(14)_ = 1.72, *p* = 0.108) or absence (Figure 4; *t*_(16)_ = 1.05, *p* = 0.307) of EE.

## Discussion

Our results show that DBS of ventromedial prefrontal cortex consistently produces anxiolysis when animals are housed in EE, but not when animals are housed in standard laboratory housing. For example, DBS animals exhibited more robust exploration of anxiogenic parts of elevated plus-maze compared with sham-stimulated animals. Yet this was true only if animals were housed in EE and not in standard housing environment. It is noteworthy that mean anxiety score of sham animals in standard housing was much lower than sham animals living in enriched housing. Thus, lack of DBS effect in sham animals might have been influenced by a floor effect of very low anxiety in control animals. Similarly, DBS-treated animals exhibited more active defense response in the plus-maze characterized by risk assessment rather than avoidance. This effect was also evident only when animals were housed in EE and not when animals were placed in standard non-EE conditions. These observations suggest that the complexity of housing environment is an important determinant of therapeutic outcome of DBS treatment in the context of emotional behaviors. This is further buttressed by comparisons of magnitude of DBS effects in the two housing environments. Effect size of DBS on anxiety-related endpoints was very robust with *d* values from 1.0 to 1.4 when housed in the EE. In contrast, DBS exhibited statistically significant effect in only one endpoint, and the observed effect size was weaker in that case (*d* = 0.8). To keep this in numerical perspective, a randomly chosen EE-housed DBS-treated subject had >75% probability of showing lower emergence latency from home-cage when compared with a randomly chosen EE-housed sham-treated animal. Similarly, a randomly chosen EE-housed DBS-treated animal had >83% probability of making more active risk assessment maneuvers compared with a randomly chosen EE-housed sham animal. In contrast, DBS induced anxiolysis did not reach statistical significance in elevated plus-maze and exhibited a mediocre effect size of 0.8 in case of home-cage emergence latency. This suggests that DBS might be more effective for emotional disorders if used in conjunction with environmental interventions. Effects of DBS and its higher efficacy with EE showed specificity to the anxiety-related endpoints, with nondiscernible effects on nonemotional object-recognition task.

The EE, in this study, comprises several social and nonsocial facets. For example, standard housing in our design entails housing animals singly after the surgery due to possibility of damage to the electrodes. Animals in the enriched housing, meanwhile, stay in a social setting of four animals per larger cage during the similar time window. This could have changed the social landscape of the housing environment including social interaction and dominance relationships. This is relevant because single housing is known to induce anxiety in rats (Balcombe, 2006). Our study design does not allow statistical comparisons between sham-treated animals living in standard housing vs. enriched housing, due to the orthogonal nature of the planned analysis. The enriched housing also consisted of changes in nonsocial aspects of the environment including greater availability of sensory stimuli and exposure to novelty. Our results cannot determine if greater efficacy of DBS in EE animals was due to social factors, nonsocial factors, or their emergent interaction.

Several studies suggest that DBS has the potential to manage fear and anxiety-related behaviors in preclinical animal models. For example, DBS of basolateral amygdala reduces anxiety when measured in a defensive burying task, but not when measured in elevated plus-maze (160 Hz for 4 h per day for 7 days; Langevin et al., 2010; Stidd et al., 2013). Similar stimulation paradigm also decreases the strength of fear conditioning to a discrete auditory tone, but not the contextual fear conditioning (200 Hz for 4 h per day for 7 days; Sui et al., 2014). The DBS effects in ventromedial prefrontal cortex show similar equivocality in experiments reported here. The DBS-treated animals in standard housing regimes exhibit anxiolysis when measured in the home-cage emergence task, but not in elevated plus-maze. It is plausible that ambivalence in DBS effects in these studies is an artifact of the impoverished housing environment in standard laboratory practice.

Several EE paradigms have been previously used to show the beneficial effects of complex housing on emotional behaviors in animal models. For example, peripubertal EE reduces anxiety and depression (Francis et al., 2002; Cui et al., 2006; Ilin and Richter-Levin, 2009). Similarly, EE provided in adulthood reduces anxiogenesis brought about by historical stress exposure (Koe et al., 2016; Ashokan et al., 2018a). These studies have led to an emphasis on the critical role of living environment and potential to exhibit species-typical behaviors for emotional well-being. Our studies advance this by showing that EE can facilitate beneficial effects of a targeted and intensive surgical intervention such as DBS.

The DBS is a very appropriate exemplar of how animal studies can lead to clinical outcomes. Use of DBS is now mainstream for movement disorders like Parkinson’s disease. The impetus for this adoption comes from carefully controlled animal studies, showing that stimulation of basal ganglia paradoxically had the same effect as lesions: which is to reduce motor symptoms in an animal model of the Parkinson’s. This knowledge in animals directly led to experimental use of DBS in human patients. Observations in this report further that narrative by suggesting intimate interplay of DBS and ambient environment, whereby, environment enrichment enhances behavioral plasticity brought about DBS.

## Author Contributions

RM conceptualized, planned, analyzed and wrote the manuscript. YB and LL planned, executed and analyzed the data.

## Conflict of Interest Statement

The authors declare that the research was conducted in the absence of any commercial or financial relationships that could be construed as a potential conflict of interest.
